# Functionality of the putative surface glycoproteins of the Wuhan spiny eel influenza virus

**DOI:** 10.1038/s41467-021-26409-2

**Published:** 2021-10-25

**Authors:** Guha Asthagiri Arunkumar, Disha Bhavsar, Tiehai Li, Shirin Strohmeier, Veronika Chromikova, Fatima Amanat, Mehman Bunyatov, Patrick C. Wilson, Ali H. Ellebedy, Geert-Jan Boons, Viviana Simon, Robert P. de Vries, Florian Krammer

**Affiliations:** 1 Department of Microbiology, Icahn School of Medicine at Mount Sinai, New York, NY USA; 2 Graduate School of Biomedical Sciences, Icahn School of Medicine at Mount Sinai, New York, NY USA; 3 Complex Carbohydrate Research Center, University of Georgia, Athens, GA USA; 4 Department of Chemical Biology and Drug Discovery, Utrecht University, Utrecht, Netherlands; 5 Departmentof Medicine, University of Chicago, Chicago, IL USA; 6 Department of Pathology and Immunology, Washington University School of Medicine, St. Louis, MO USA

**Keywords:** Influenza virus, Viral membrane fusion

## Abstract

A panel of influenza virus-like sequences were recently documented in fish and amphibians. Of these, the Wuhan spiny eel influenza virus (WSEIV) was found to phylogenetically cluster with influenza B viruses as a sister clade. Influenza B viruses have been documented to circulate only in humans, with certain virus isolates found in harbor seals. It is therefore interesting that a similar virus was potentially found in fish. Here we characterize the putative hemagglutinin (HA) and neuraminidase (NA) surface glycoproteins of the WSEIV. Functionally, we show that the WSEIV NA-like protein has sialidase activity comparable to B/Malaysia/2506/2004 influenza B virus NA, making it a *bona fide* neuraminidase that is sensitive to NA inhibitors. We tested the functionality of the HA by addressing the receptor specificity, stability, preferential airway protease cleavage, and fusogenicity. We show highly specific binding to monosialic ganglioside 2 (GM2) and fusogenicity at a range of different pH conditions. In addition, we found limited antigenic conservation of the WSEIV HA and NA relative to the B/Malaysia/2506/2004 virus HA and NA. In summary, we perform a functional and antigenic characterization of the glycoproteins of WSEIV to assess if it is indeed a *bona fide* influenza virus potentially circulating in ray-finned fish.

## Abstract (web summary)

The recently identified Wuhan spiny eel influenza virus (WSEIV) sequence is more closely related to influenza B than A viruses. Here, the authors functionally characterize the putative surface glycoproteins of WSEIV and show that its NA-like protein has sialidase activity and its HA-like protein binds monosialic ganglioside 2.

## Introduction

Influenza A and B viruses cause widespread infections in humans on an annual basis resulting in significant morbidity and mortality^1^. In addition to human infections, influenza A virus has been shown to have a broad host tropism, infecting a variety of different avian and mammalian species^2^. This enhances the pandemic potential of these viruses due to an increased possibility of reassortment in a commonly infected host^3^. In contrast, influenza B viruses have a comparatively limited host range comprising predominantly of infections in humans. Sporadic outbreaks have been observed in harbor seals and gray seals, and this has brought into question the possibility of non-human reservoirs for influenza B viruses^4,5^. However, sequence analysis of influenza B virus isolates from seals suggests that the causative agents were human strains^6^. Partial sequences of isolates of influenza B viruses identified in swine farms across the USA displayed high homology to human influenza B virus isolates as well^7^. Overall, studies have documented spillage of influenza B virus infections from humans to other species, however, there is limited evidence for the existence of sustained animal reservoirs for these viruses.

The overall understanding of RNA virus diversity outside of avian and mammalian species has been limited stemming from sampling biases towards these hosts^8^. A study by Shi et al. aimed at addressing this dearth of sampling in amphibians, fish, and reptiles, and identified 214 vertebrate species-associated virus sequences through a meta-transcriptomic approach. Most of these viruses could be categorized into 17 vertebrate-specific viral families, significantly enhancing the diversity of viral families historically known to have mammalian or avian hosts. Three novel influenza viruses were identified in ray-finned fish (spiny eel), jawless fish (hagfish), and amphibians (Asiatic toad). These were the first documented sequences of putative influenza viruses in fish^9^.

For the Wuhan spiny eel influenza virus (WSEIV), all eight genomic segments were recovered following the sampling and analysis of the transcripts in the gill tissues of lesser spiny eels (*Macrognathus aculeatus*). A striking aspect of this virus is its phylogenetic clustering as a sister clade to influenza B viruses, more so than influenza A viruses do. Alignment of the coding regions of the eight segments of WSEIV indicates percentage identity as high as 76% (PB1) and as low as 34% (NS) with the closest hits all being in the influenza B virus species. The surface viral glycoproteins, hemagglutinin (HA), and the neuraminidase (NA) of the WSEIV have a 45% and 48% amino acid identity to the respective HA and NA of influenza B viruses^9^.

Given our interest in studying viral glycoproteins, we decided to take a deeper dive into characterizing the HA and NA of the WSEIV and their influenza B virus counterparts. This could provide valuable insight into this novel influenza B-like virus, and its implication of non-human reservoirs for influenza B viruses. Additionally, influenza B viruses are largely understudied relative to influenza A viruses in the context of the functionality and antigenic landscape, and the findings from this study contribute towards addressing this gap^10^. The questions raised during the conception of this study were as follows. Does the WSEIV HA have sialic acid-binding activity and what receptor specificity does it possess? Does the WSEIV neuraminidase have sialidase enzymatic activity? How do the functional and antigenic aspects of these proteins compare to those of influenza B virus HAs and NAs and how does this inform the potential of this virus to spill over into humans?

Here, we demonstrate that the WSEIV HA and NA show opposing similarity profiles relative to influenza B/Malaysia/2506/2004 virus HA and NA. The WSEIV HA appears to strongly interact with a unique gangliosidic receptor, displaying drastically different target receptor specificity compared to influenza B virus HAs. Additionally, we show that the WSEIV HA can be activated by trypsin, but not any human airway proteases, and it is fusogenic. Taken together, the WSEIV HA interacts with a sialic acid-containing receptor, can be activated by a protease, and can mediate fusion. On the contrary, the WSEIV NA, showing sialidase activity, has a notably similar activity profile relative to the control influenza B virus NA. Cleavage of the sialic acid residue on this gangliosidic receptor by the WSEIV NA was also confirmed. Additional functional characterization further reinforces this dichotomous nature of the HA and NA. Overall, we show that this WSEIV is indeed a *bona fide* influenza virus from a functional standpoint. We also address the antigenic conservation of the WSEIV HA and NA using a panel of broadly cross-reactive monoclonal antibodies (mAbs) and a set of serum samples from humans positive for the influenza B virus.

## Results

**Fig. 1:**
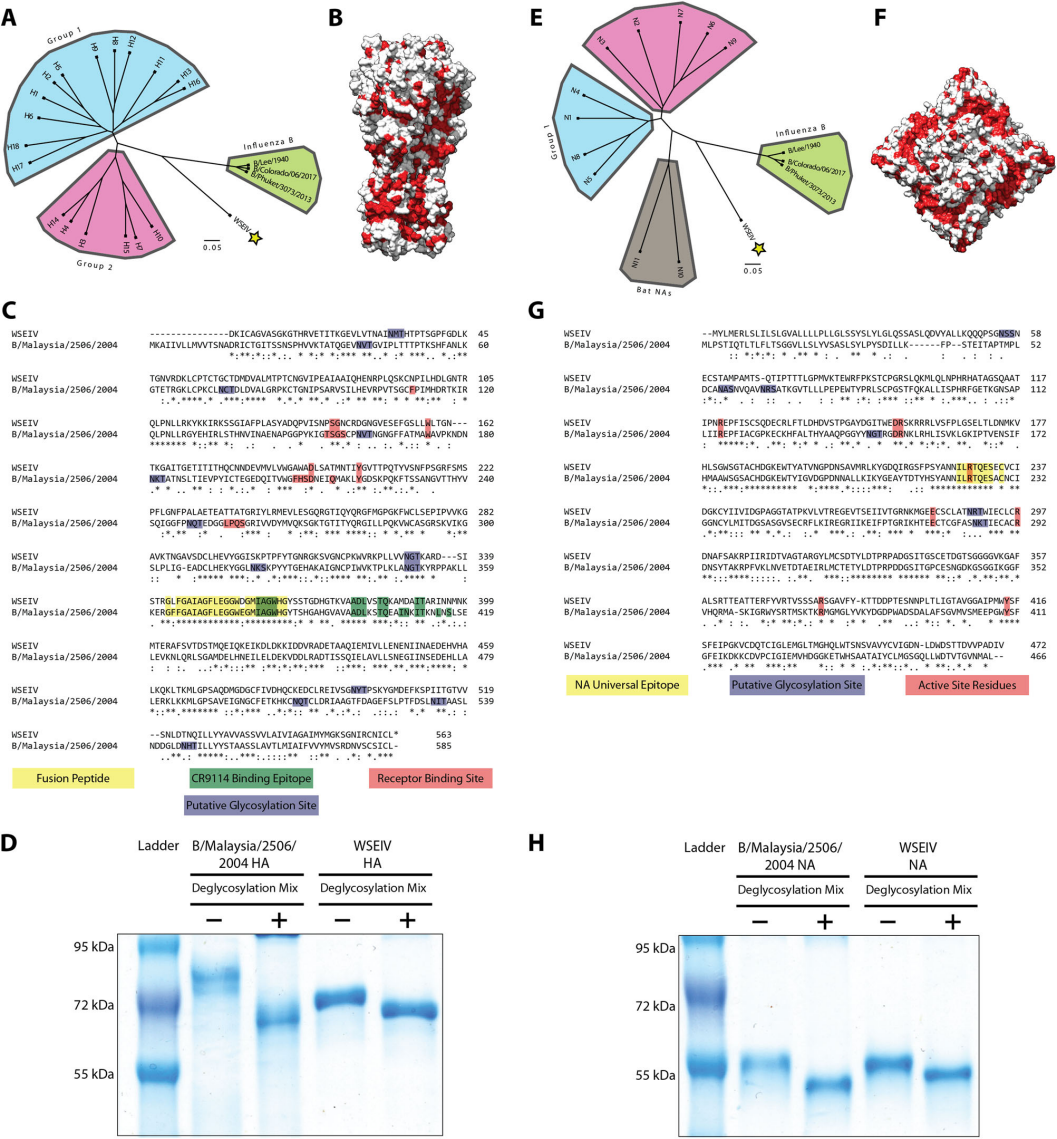
Comparative sequence analysis of the WSEIV glycoproteins. Phylogenetic trees based on the amino acid sequences of the WSEIV glycoproteins and the representative influenza A and B HAs (**A**) and NAs (**E**) obtained from GISAID are shown. The scale bar indicates a 5% difference in amino acid sequence. Amino acid conservation of the WSEIV HA (**B**) and WSEIV NA (**F**) relative to influenza B virus HA and NA are represented by the residues highlighted in red. The structure and sequence of influenza virus strain B/Brisbane/60/2008 were used as a template for comparison. Pairwise alignments of the WSEIV HA and NA are displayed against influenza B/Malaysia/2506/2004 virus HA and NA, (**C**) and (**G**), respectively. Identical residues are indicated by asterisks. Functionally and antigenically relevant features have been annotated. Recombinantly expressed HAs (**D**) and NAs (**H**) of influenza B/Malaysia/2506/2004  virus and WSEIV were visualized on an SDS–PAGE gel in deglycosylated and non-deglycosylated conditions. A representative of two independent assays is shown. Structures are based on PDB# 4FQM^50^ for HA and PDB# 4CPL^51^ for NA.

Representative HA and NA amino acid sequences of influenza A and B viruses were selected and phylogenetically compared to the WSEIV HA and NA. Along the lines of the whole virus genome alignments in the study identifying this virus, we observed the proximal clustering of the WSEIV HA and NA to influenza B virus HAs and NAs (Fig. 1A, E). This encompasses the seasonal vaccine strains from the two influenza B virus antigenic lineages, B/Victoria/2/1987-like and B/Yamagata/16/1988-like, and the ancestral pre-divergence B/Lee/1940 virus too^11^. The sequences of each glycoprotein were superimposed onto the publicly available structure of influenza B/Brisbane/60/2008 virus counterparts to visualize where the ~45% (HA) and ~48% (NA) identity is present (Fig. 1B, F). As a comparative control, influenza B/Malaysia/2506/2004 (part of the B/Victoria/2/1987-like lineage) virus was selected and the HA and NA of this virus were used for the experiments detailed in this study. These glycoproteins have previously been expressed in our laboratory at high yields and purity. We are also equipped with a mouse-pathogenic B/Malaysia/2506/2004 challenge virus and reverse genetics system for possible future research avenues to study the WSEIV HA and NA. A pair-wise alignment of the WSEIV HA and NA with the influenza B/Malaysia/2506/2004 virus HA and NA was carried out (Fig. 1C, G). In the context of the HA, there appeared to be mismatches in the residues that constitute the sialic acid interacting receptor binding site^12,13^. This lack of conservation was indicative of a potentially altered receptor binding profile of the WSEIV HA. Additionally, the WSEIV HA also appeared to have a reduced number of putative N-linked glycosylation sites as identified by the consensus sequence N-X-(S/T). The reduced glycosylation on the WSEIV HA relative to the influenza B/Malaysia/2506/2004 HA was confirmed using a deglycosylation assay with the respective recombinant proteins. A larger difference in molecular weight was observed on a reducing sodium dodecyl sulfate–polyacrylamide gel electrophoresis (SDS–PAGE) gel for the B/Malaysia/2506/2004 HA pre- and post-deglycosylation in comparison to the WSEIV HA (Fig. 1D). From an antigenic standpoint, the target epitope of the pan-influenza virus HA mAb, CR9114, was found to have some mismatches too^14^. A large number of matched residues, however, appear to be in the stalk domain of the HA, consistent with previous studies showing higher levels of conservation in this region across all influenza virus HAs^15^. Significant mismatches were observed in the region immediately upstream to the fusion peptide, largely conserved, encompassing the proteolytic cleavage site essential for activation of the HA. The comparison of the NA sequences, on the other hand, demonstrated a conserved enzymatic active site, as are the regions in its immediate vicinity. A deglycosylation analysis of the B/Malaysia/2506/2004 and WSEIV NA did not reveal any overt differences in glycosylation patterns across the glycoproteins with comparable shifts in molecular weights pre- and post-deglycosylation (Fig. 1H).

The WSEIV HA and NA were studied and characterized in the form of recombinant proteins as opposed to in a viral backbone for several reasons. The available sequences for these proteins comprised only the coding regions from the study identifying them. Consequently, the packaging sequences in the non-coding regions essential to rescuing these glycoproteins in an influenza B virus backbone were not available. More importantly, introducing novel glycoproteins into a known human pathogen, e.g. a human-adapted influenza B virus backbone possesses a biosafety risk. As a result, these proteins were studied in a recombinant form to ensure to meet safety concerns. The B/Malaysia/2506/2004 and WSEIV HAs and NAs were expressed using a baculovirus expression system as previously described^16^. Since the available sequence for the WSEIV HA consisted of a truncated signal sequence, a full-length signal peptide from the B/Malaysia/2506/2004 virus HA was added instead. A C-terminal T4 trimerization domain was used to ensure the expression of the HAs in their native trimeric state. An N-terminal vasodilator stimulating phosphoprotein (VASP) tetramerization domain was used to maintain tetrameric structures of the respective neuraminidases.

**Fig. 2:**
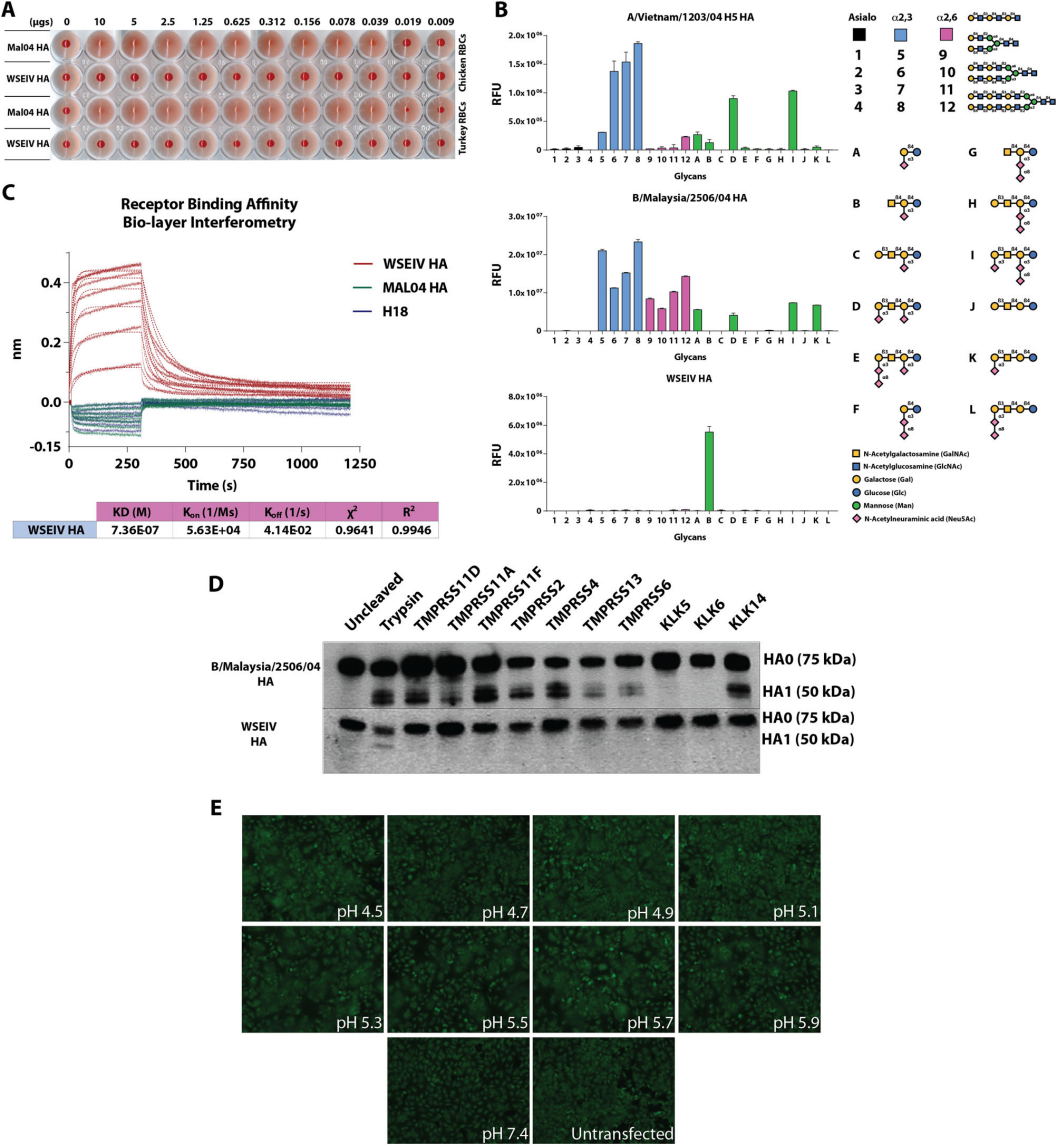
The WSEIV HA has a divergent functional profile relative to the influenza B/Malaysia/2506/2004 virus HA. **A** Recombinant HA proteins starting at 10 µg serially diluted two-fold were applied in a classical hemagglutination assay with 0.5% chicken or turkey erythrocytes. **B** Receptor binding specificity of the WSEIV HA was determined using a glycan microarray. A/Vietnam/1204/2004 H5 and B/Malaysia/2506/2004 HAs served as controls to compare binding profiles of the HAs. Glycans 1-4 represent asialic structures with the indicated backbones. Glycans 5-8 and 9-12 are α2,3- and α2,6-linked sialic acid glycans, with A–L comprising complex glycans and gangliosides. A simplified structure of the GM2 ganglioside, sample B on the array is shown. The mean signal for each glycan–HA binding is shown with each glycan printed in replicates of 6. Error bars indicate standard deviation and technical replicates were performed. The binding was confirmed with two separate batches of recombinant WSEIV HA. **C** The WSEIV HA–GM2 ganglioside interaction was validated by bio-layer interferometry. B/Malaysia/2506/2004 HA and H18 HA were used as negative controls with expected absence of binding towards GM2. The association and dissociation kinetics curve fits of the interaction are shown and the dissociation constant (*K*_d_) was calculated accordingly. **D** Western blotting was performed to determine the proteolytic cleavage profile of the WSEIV and B/Malaysia/2506/2004 HAs by human airway proteases. Cell lysate was generated following co-transfection with pCAGGS HAs and pcDNA3.1 protease expressing plasmids. HA1 was detected as a marker for proteolytic cleavage alongside uncleaved HA0. Cells transfected with pCAGGS HA either untreated or exposed to TPCK-treated trypsin served as controls for proteolytic cleavage. Representative images of two independent assays are shown. **E** A polykaryon formation assay was applied to determine the fusogenicity of the WSEIV HA. HeLa cells transfected with the pCAGGS WSEIV HA were treated with TPCK-treated trypsin, exposed to different indicated pH conditions, and allowed to recover to determine for cell-to-cell fusion. This assay was performed in independent duplicates and a representative image is shown.

To determine whether the WSEIV HA is capable of hemagglutinating erythrocytes, a conventional hemagglutination assay was carried out. Recombinant WSEIV and B/Malaysia/2506/2004 HA were incubated with 0.5% chicken and turkey erythrocytes starting at a concentration of 10 µg of recombinant HA. The B/Malaysia/2506/2004 HA caused hemagglutination of both chicken and turkey RBCs while an absence of hemagglutination was observed with the WSEIV HA at  the same concentrations (Fig. 2A). Although this has been observed for recent H3N2 virus isolates, we were intrigued by the lack of hemagglutination and questioned the receptor usage of the WSEIV HA^17^. The receptor binding specificity of the hemagglutinin protein of influenza viruses is a vital parameter in addressing the potential of these viruses to cross species barriers and determining host and tissue tropism for infection^18^. Glycan arrays are an instrumental tool in identifying the target receptor specificity for influenza virus HAs in determining whether they preferentially bind to α2,3-linked or α2,6-linked sialic acid receptors. We took advantage of this approach to further interrogate interactions between WSEIV HA and its potential binding partners. As a control, the recombinant H5 HA from A/Vietnam/1203/2004 H5N1 influenza A virus, influenza B/Malaysia/2506/2004 virus rHA, and the WSEIV rHA were applied to a glycan array comprising of a variety of asialo, α2,3-linked, α2,6-linked and gangliosidic structures (Fig. 2B). All recombinant HAs were probed with a fluorescently tagged anti-hexahistidine antibody to determine the extent of binding to different glycans present on the array. In line with previously published receptor specificity profiles for avian HAs, the H5 rHA preferentially and predominantly binds to α2,3-linked sialic acids present on the array^19^. The rHA of influenza B/Malaysia/2506/2004 virus shows binding to both α2,3-linked and α2,6-linked sialic acids, which has previously been attributed to the presence of a Phe95 residue in influenza B virus HA as opposed to a conserved tyrosine residue at the same site in influenza A viruses^20,21^. The WSEIV HA showed a unique binding profile on the glycan array (Fig. 2B), no binding was observed to α2,3-linked or α2,6-linked sialic acids of increasing length as seen for the influenza B virus HA. Strong fluorescence intensity indicative of binding was observed on a singular spot on the array corresponding to a ganglioside oligosaccharide, GM2. This monosialylated ganglioside has an α2,3-linked sialic acid at the penultimate galactose residue. To validate this interaction of the WSEIV HA and GM2, bio-layer interferometry was applied to determine a dissociation constant (*K*_d_) and thereby binding affinity (Fig. 2C). To do so, Ni-nitriloacetic acid (Ni-NTA) sensors were loaded with fixed concentrations of hexahistidine-tagged rHA proteins (10 µg/ml) and dipped in 1.5× fold serial dilutions of recombinant GM2 starting at 100 µM. After ensuring that the sensors were loaded to saturation with the hexahistidine-tagged HAs, the association and dissociation kinetics of the HA–GM2 interaction were studied. An A/flat-faced bat/Peru/033/10 H18 hemagglutinin was applied as a negative control given its unconventional nature to interact with MHC class II as receptors for entry as opposed to sialic acid residues^22^. No binding was observed for the H18 rHA and as observed previously in the glycan array, B/Malaysia/2506/2004 rHA also showed no binding to GM2. The WSEIV HA on the other hand shows excellent association and dissociation profiles in a biphasic fashion with GM2 in a dose-dependent manner. Analysis of this binding allowed us to determine the *K*_d_ value of this interaction at 7.36 × 10^−7^ M, in the micromolar range, with *R*^2^ and *χ*^2^ indicating good curve fits. These findings indicate and validate that the GM2 ganglioside is a target receptor for this WSEIV HA. Evidently, the WSEIV HA has a contrasting sialic acid-binding profile in comparison to the influenza B virus rHA control.

Typically, following the receptor interaction of the HA with sialic acid, the HA mediates membrane fusion of the influenza virion allowing for the subsequent steps of infection and replication to occur. However, this requires the HA to be fusion competent, a state that is reliant on host cell protease-mediated activation. Cleavage by host proteases at the target site on the HA upstream of the fusion peptide splits the HA0 precursor into the HA1 and HA2 polypeptides^23^. Recently, a study dissected the difference in preferential proteolytic cleavage of influenza A and influenza B virus HAs by human airway proteases^24^. This is crucial in addressing host adaptation of HAs and instrumental in the crossing of species barriers with relative ease as observed with highly pathogenic avian influenza viruses containing a polybasic cleavage site, resulting in easier cleavage and priming of the HA by furin-like proteases^25^. Influenza B virus HAs have been found to be cleaved by a broad range of human airway proteases belonging to the type II transmembrane serine protease and kallikrein protease families^24^. We assessed the extent to which these proteases can cleave the WSEIV HA (Fig. 2D). Human embryonic kidney (HEK) 293T cells were co-transfected with pCAGGS expression plasmids encoding for either the WSEIV or the B/Malaysia/2506/2004 HA and pcDNA expression plasmids encoding individual human airway proteases. The proteases selected here have been previously shown to cleave influenza B virus HAs^24^. As an untreated control, HEK293T cells were transfected only with pCAGGS expression plasmids encoding the respective HAs. N-tosyl-l-phenylalanine chloromethyl ketone (TPCK)-treated trypsin was used as an additional control, with pCAGGS HA-transfected cells being incubated with exogenous trypsin briefly prior to harvesting of the cells. Cleavage of HA0 was detected through Western blotting after running the transfected cell lysate on a reducing sodium dodecyl sulfate–polyacrylamide gel electrophoresis (SDS–PAGE) gel. A pool of monoclonal antibodies that are broadly cross-reactive to influenza B virus HAs and polyclonal sera raised against the WSEIV rHA in mice were used to detect cleavage of the B/Malaysia/2506/2004 HA and WSEIV HA, respectively. Proteolytic cleavage of the B/Malaysia/2506/2004 HA was detected to varying extents with this panel of human airway proteases, evidenced by the presence of the HA1 bands (~50 kDA) in addition to the HA0 bands (75 kDA). Strikingly, none of the selected human airway proteases were able to cleave and activate the WSEIV HA. HA1 was detectable only in the trypsin treated sample (Fig. 2D). To determine whether the WSEIV HA is fusogenic, a polykaryon formation assay was performed at different pH conditions. HeLa cells were transfected with pCAGGS expressing the WSEIV HA. Following trypsin treatment to activate the HAs expressed on the cell surface, the cells were exposed to different pH conditions and incubated for 4 h to allow fusion to occur. Visualization of the cells at different pH conditions showed the presence of polykaryons at a variety of different pHs tested from pH 4.5 to 5.9 (Fig. 2E). No polykaryons were formed at a control pH of 7.4 or in untransfected cells. Overall, this result supports the notion that the WSEIV HA is functionally divergent from the influenza B virus HA. The WSEIV HA interacts with a unique GM2 gangliosidic receptor, is not activated by human airway proteases, and is fusogenic at a wide range of pH conditions.

**Fig. 3:**
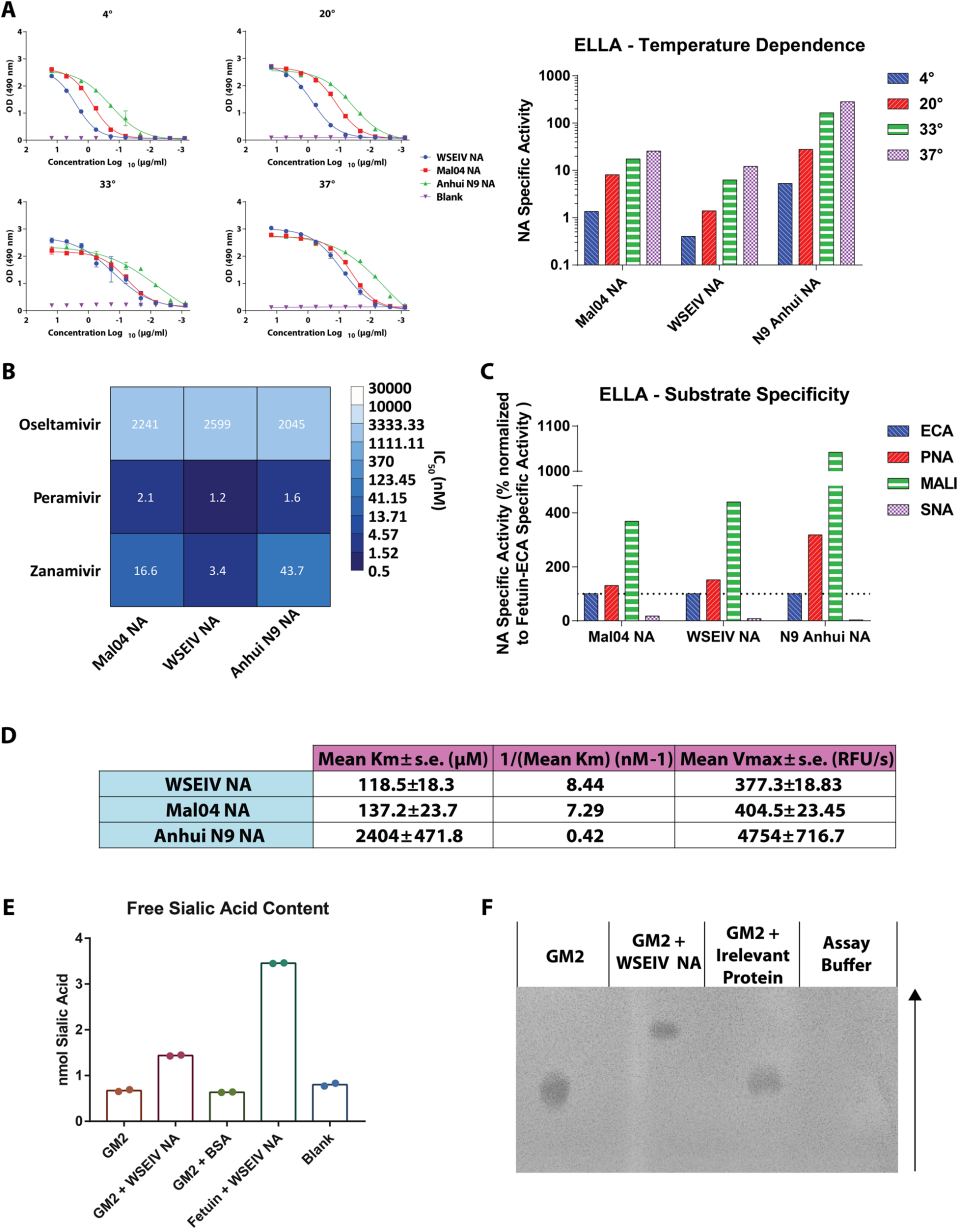
Functionally, the WSEIV NA is strikingly similar to the influenza B/Malaysia/2506/2004 virus NA. **A** Sialidase activity of recombinant WSEIV, influenza B/Malaysia/2506/2004 and A/Anhui/1/2013 H7N9 NA proteins was evaluated in an enzyme-linked lectin assay using fetuin, at four temperatures; 4, 20, 33, 37 °C. The curves indicate absorbance measured at 490 nm with error bars indicating standard deviation. Specific enzyme activity (inverse of half-maximum lectin binding) determined by these absorbance curves are shown for each individual NA at each temperature. **B** Susceptibility to NA inhibitors was tested in an ELLA-based neuraminidase inhibition assay . Error bars indicate standard deviation. Absorbance at 490 nm based on lectin binding was measured with increasing concentrations of oseltamivir, peramivir or zanamivir. **C** Neuraminidase substrate specificity was assessed using an ELLA with fetuin and lectins with different specificities (ECA, PNA, SNA, and MALI). Specific enzyme activity was determined as described in (**A**) and it is represented as a percentage normalized to fetuin-ECA. **D** Michaelis–Menten parameters, V_max_ and K_m_, were estimated based on enzymatic activity of recombinant NAs against MUNANA substrate. The A/Anhui/1/2013 N9 NA served as a comparative control for both the WSEIV and B/Malaysia/2506/2004 NAs. **E** Cleavage of sialic acid on GM2 by the WSEIV NA was confirmed using an assay to measure free sialic acid content (sialic acid NANA assay kit, Abcam). **F** Thin layer chromatography was used to visualize the presence of cleaved GM2 following treatment with the WSEIV NA. The arrow shows the direction in which the chromatography was run. A representative image is shown after the assay was performed in independent duplicates. Technical duplicates were performed once for (**A**–**E**).

Given that the primary active site residues crucial for the sialidase activity of the NA are conserved, we tested the functional activity of the WSEIV NA. To do so, a conventional enzyme-linked lectin assay (ELLA) was carried out using fetuin as a substrate (Fig. 3A). Recombinant NAs were applied in 3-fold serial dilutions starting at 15 µg/ml and the extent of NA activity was determined based on the extent of sialic acid cleavage, detected by binding of peanut agglutinin lectin to exposed galactose residues. The overnight incubation of the rNA with fetuin was carried out at four different temperatures, 4, 20, 33, and 37 °C to determine the temperature dependence of the NA activity. These were selected to encompass the possible temperatures encountered by the spiny eels in their freshwater environment in the Wuhan area. Given that the enzymatic activity of the influenza B NA is understudied, an N9 rNA from the A/Anhui/1/2013 H7N9 virus was used as a comparative control. The major observation was that the WSEIV NA does indeed have neuraminidase activity (Fig. 3A). This enzymatic activity is also starkly similar to that of the B/Malaysia/2506/2004 NA at the tested temperatures. Preliminary analysis suggests that the N9 rNA appears to have higher enzymatic activity compared to both the influenza B virus and influenza B virus-like NAs. To better understand the temperature dependency, the specific activity of the NA was determined using the inverse of the half-maximum lectin binding. As expected, we observe a step-wise reduction in the activity of each NA at lower temperatures. The specific activity profile is also almost identical for the WSEIV NA and the B/Malaysia/2506/2004 NA.

Keeping in mind the novel aspect of the WSEIV NA recently identified in a non-human host, we investigated its potential sensitivity to the NA inhibitor such as oseltamivir (Tamiflu), peramivir or zanamivir (Fig. 3B). This was tested using a neuraminidase inhibition ELLA assay wherein fixed concentrations of rNAs were pre-incubated with oseltamivir starting at 156.28 µM and serially diluted two-fold prior to being transferred onto fetuin coated plates. The extent of inhibition was estimated by increased lectin binding with lower oseltamivir concentrations. The WSEIV NA was sensitive to oseltamivir, and the dose dependency was identical for all the tested recombinant NAs (Fig. 3B). Similar results were obtained with peramivir and zanamivir although these drugs showed higher potency than oseltamivir. These findings also align with the aforementioned observation that the active site of the WSEIV NA and the surrounding regions are well conserved to the B/Malaysia/2506/2004 NA allowing for the binding and inhibition by NA inhibitors.

To follow up on the unique receptor binding profile shown by the WSEIV HA, we questioned whether the WSEIV NA has altered substrate specificity compared to the B/Malaysia/2506/2004 NA (Fig. 3C). To probe this, ELLAs were carried out as previously described in a study looking at N9 NA in H7N9 influenza A viruses^26^. Lectins with different binding specificities were used to determine if the NAs preferentially cleaved α2,3-linked or α2,6-linked sialic acids using fetuin as a substrate which carries both these linkages in a 2:1 ratio^27^. Specifically, *Erythrina crista-galli* (ECA), peanut agglutinin (PNA), *Maackia amurensis* lectin I (MALI), and *Sambucus nigra* lectin (SNA) were used. ECA and PNA both have been found to show binding to non-siaylated N- and O-linked sugars respectively, hence their binding would be higher in the presence of a NA^28,29^. MALI and SNA preferentially bind only α2,3- and α2,6-linked sialic acids, respectively, and their binding decreases with NA cleavage of the target substrates^30,31^. As described earlier, the specific activity of the NA was determined and normalized to that seen with fetuin-ECA for each NA. In line with the enzymatic activity, the WSEIV and B/Malaysia/2506/2004 NA have equivalent substrate specificities, with both having comparable cleavage profiles (Fig. 3C). Although a similar conclusion can be drawn for the A/Anhui/1/2013 N9 neuraminidase, this preferential cleavage appears to be more polarized. Subsequently, we characterized the kinetics of the enzymatic reactions of these NAs, i.e. do these NAs have comparable enzyme kinetics despite having similar temperature-dependent activity and substrate specificity? To investigate this, we derived the Michaelis–Menten parameters, *V*_max_ and *K*_m_, from the NA–sialic acid enzyme–substrate reaction (Fig. 3D). At a fixed concentration, rNAs were incubated with the fluorogenic 4-methylumbelli-feryl N-acetyl-α-d-neuraminic acid (MUNANA) substrate and the relative fluorescence readings were taken every 90 s for 40 min as previously described^32^. The velocity of the reactions were determined for each concentration of MUNANA (starting at 1000 µM) and accordingly the *V*_max_ and *K*_m_ were calculated. The maximum enzymatic activity, *V*_max_, for both the WSEIV and B/Malaysia/2506/2004 NA are similar to each other, and they both display a high affinity for the MUNANA substrate (indicated by the 1/*K*_m_). The N9 rNA shows exponentially higher maximum enzymatic activity and a markedly lower affinity for the substrate, which could possibly explain the stronger specific activity seen earlier while measuring substrate affinity.

The neuraminidase plays a crucial role in the viral life cycle in the context of facilitating viral egress and release through the cleavage of sialic acid bound to the viral HA. This allows for productive infection cycles in the host. In context of WSEIV, this would require the NA-mediated cleavage of the α2,3-linked sialic acid residue on the cognate GM2 receptor of the WSEIV HA. To determine whether the WSEIV NA possesses this ability, a free sialic acid assay and a thin layer chromatography-based approach were used in parallel. Recombinant GM2 was incubated with the WSEIV NA and the free sialic acid content was evaluated using a commercially available kit. As a negative control, GM2 was incubated with an irrelevant protein (bovine serum albumin). Fetuin incubated with WSEIV NA was used as a positive control. Cleavage of the sialic acid on GM2 was observed as determined by the presence of free sialic acid (Fig. 3E). This was also visualized following thin-layer chromatography wherein GM2 incubated with WSEIV NA ran further on the chromatogram in comparison to untreated GM2 or GM2 treated with BSA (Fig. 3F). Fetuin incubated with WSEIV NA was not visualized on this chromatogram given the higher molecular weight of fetuin compared to GM2, and hence the lack of migration following sample spotting.

**Fig. 4:**
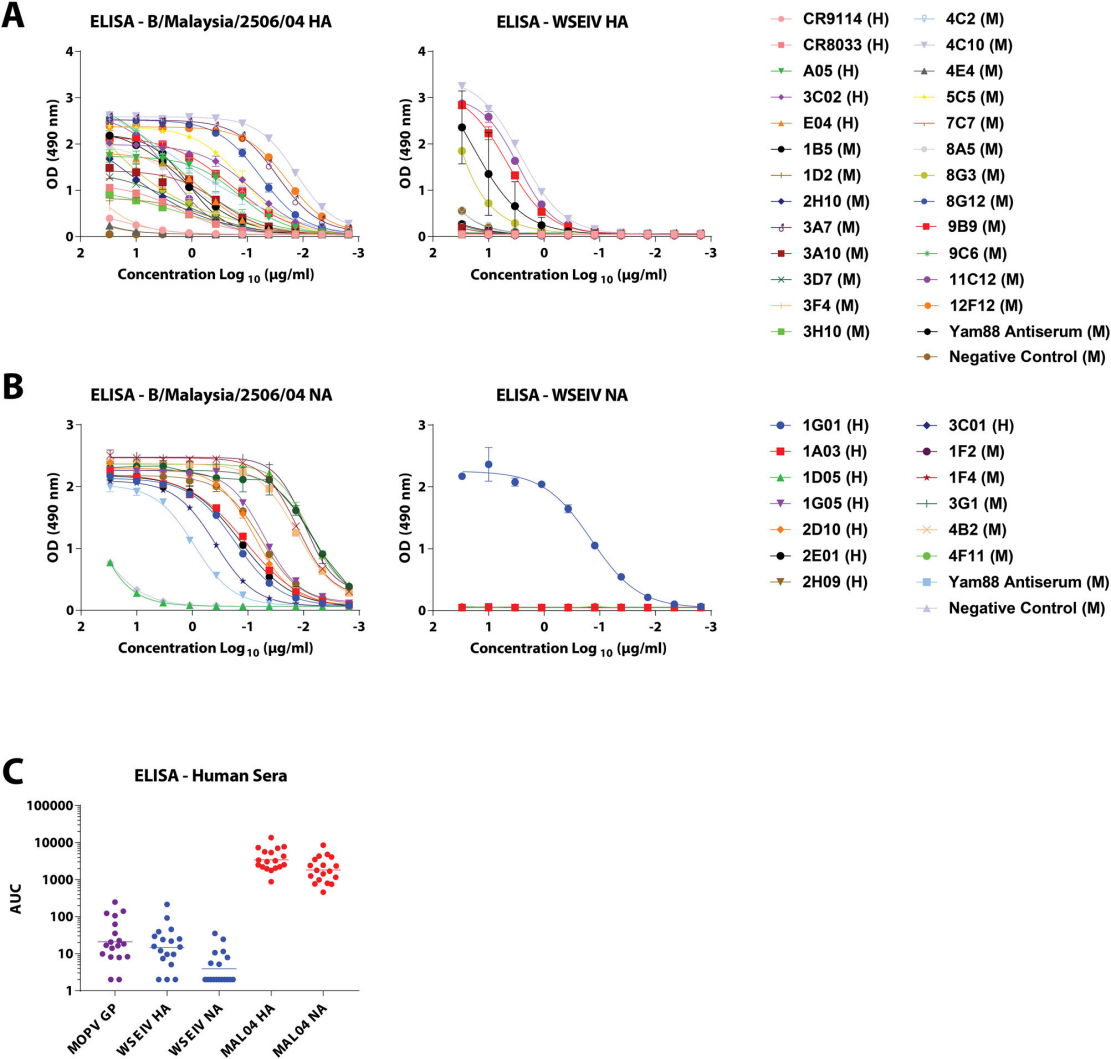
Epitopes that are broadly conserved in influenza B virus glycoproteins are largely absent the WSEIV HA and NA, with cross-reactivity being mostly restricted to the stalk domain of the HA and active site of the NA. **A** and **B** Binding profiles of broadly cross-reactive anti-influenza B virus HA and NA human (H) and mouse (M) monoclonal antibodies in ELISAs are shown against recombinant B/Malaysia/2506/2004 and WSEIV HA and NAs. 4F11 (anti-influenza B virus NA) and 4C2 (anti-influenza B virus HA) mouse mAbs were used as negative controls for the ELISAs against the HAs and NAs respectively. **C** Presence of pre-existing immunity or induction of cross-reactive antibodies against the WSEIV HA and NA was evaluated through ELISAs using human serum samples obtained post-seasonal influenza vaccination (*n* = 18). Mopeia virus glycoprotein was used as a negative control for baseline establishment, and area under the curve was calculated with a cutoff of average plus three times the standard deviation of the blank wells. The geometric mean for each group is indicated with a line. Experiments were performed once in technical duplicates.

Having characterized the functionality of the WSEIV HA and NA, we focused on surveying the extent to which broadly cross-reactive epitopes identified on influenza B virus HAs are conserved on these glycoproteins (Fig. 4A, B). Probing of the WSEIV HA and NA was carried out using a panel of broadly cross-reactive human and mouse monoclonal antibodies (mAbs) previously characterized to bind to both antigenic divergent and ancestral lineages of influenza B viruses^14,33–37^. Control enzyme-linked immunosorbent assays (ELISAs) were performed for these mAbs against the influenza B/Malaysia/2506/2004 virus HA and NA and robust binding profiles for most of the tested antibodies were observed. Five antibodies showed strong binding to the WSEIV HA, namely, 1B5, 4C10, 8G3, 9B9, and 11C12 (all murine mAbs, Fig. 4A). With the exception of 1B5, all these mAbs bind to linear epitopes on the conserved long alpha helix in the stalk domain of the influenza B virus HA^33^. As stated earlier, conservation between the WSEIV and B/Malaysia/2506/2004 HA (or influenza B virus HAs at large) is relatively high in this region (Fig. 1B, C). No binding of the pan-influenza virus HA human mAb CR9114 was detectable, as anticipated from the mismatches in the binding epitope of the antibody^14^. The binding of CR9114 to influenza B virus HA was low but detectable. Of the panel of human and mouse mAbs used to probe the WSEIV NA, only one antibody showed detectable and strong binding (Fig. 4B). This human mAb, 1G01, has been characterized to have a target binding epitope in the active site of the NA facilitated by a long heavy chain CDR3 loop. Consequently, the breadth of the antibody encompasses influenza A and B virus NAs, showing neuraminidase inhibition activity against most of the tested influenza virus NAs^35^. Overall, we observe an interesting profile for the WSEIV HA and NA from an antigenic standpoint. For a functionally dissimilar WSEIV HA, there are a larger number of conserved epitopes distributed in the stalk domain. Conversely, identical functionality seen for the WSEIV NA with B/Malaysia/2506/2004 NA is accompanied by an absence of this conservation outside of the enzymatic active site pocket (Fig. 1E, F). Additionally, serum samples from humans post-seasonal influenza vaccination (collected between 2017 and 2019) were used in ELISAs against the WSEIV HA and NA (Fig. 4C). This was done to identify basal or pre-existing cross-reactive immunity against the WSEIV HA or NA in humans as a consequence of seasonal vaccination or prior influenza virus infection. Safely assuming immunological naivety, a recombinant glycoprotein from Mopeia virus, belonging to the arenavirus family, was used as a target antigen in ELISAs to establish baseline reactivity in our assay. No pre-existing immunity or post-vaccination-induced antibodies were detected against the WSEIV HA or NA further reinforcing the limited conservation of broadly cross-reactive target epitopes as determined by the panel of mAbs earlier.

## Discussion

Here, we have characterized the putative HA and NA of the Wuhan spiny eel influenza virus, an influenza B-like virus identified via sequence analysis in lesser spiny eels. The two studies that identified the WSEIV and a salamander influenza-like virus that also clusters close to influenza B viruses discuss the occurrence of prolonged virus–host co-divergence with several host-switching events over time^9,38^. Largely, influenza B viruses have been discounted from being a pandemic threat due to the absence of an identified sustained non-human reservoir. Co-circulating influenza B viruses in humans have been shown to have reassortment potential within the two antigenic lineages, with B/Victoria/2/87-like viruses acquiring gene segments from B/Yamagata/16/88-like viruses^39^. Taken together, this strengthens the unmet need for studies characterizing novel influenza B-like viruses in undersampled hosts and subsequently understanding the functionalities of these understudied viruses. Considering that vaccine approaches towards influenza viruses predominantly target the dominant HA - and more recently the NA - surface glycoproteins, it is vital to have a comprehensive understanding of these proteins of influenza B-like viruses too^40^. In addition, our characterization of the B/Malaysia/2506/2004 HA and NA also supplements existing literature on influenza B virus glycoproteins which is finite in contrast to studies addressing influenza A viruses.

Along with showing limited antigenic conservation, we demonstrate that the WSEIV HA interacts with an a-series ganglioside, GM2, as a target receptor. Although GM2 has been identified as an interacting partner for some reoviruses and rotaviruses, it has been shown to be not recognized by influenza A viruses, with studies demonstrating that gangliosides are entirely non-essential for influenza virus entry^41–44^. As a therapeutic target, overaccumulation of GM2 on neuronal cells has been implicated in Tay Sachs and Sandhoff diseases with mutations rendering hexosaminidases non-functional^45^. GM2 overexpression is also observed in a variety of human cancers and has been linked with increased tumor angiogenesis and metastatic potential^46,47^. The WSEIV HA also appears not to bind to GM1a or GM3 as observed from the glycan array binding analysis. The receptor binding also seems to rely on the terminal GalNAc residue given that the loss of this in GM3 is accompanied by abrogation in binding. The location of the α 2,3-linked sialic acid residue in GM2 appears to be crucial as no binding is seen to a galactose extended GM1a ganglioside. From the perspective of gene therapy, having a viral glycoprotein that selectively targets this ganglioside could prove to be instrumental when pseudotyped into viral vectors for gene delivery. In an aquatic setting, GM2 has been found to be over-expressed in gills, brain, heart, and reproductive organs of fish (zebrafish) corroborating the discovery of the WSEIV in the gills of lesser spiny eels^48^. For the WSEIV NA, our findings highlight the strong conservation of enzymatic activity and kinetics, substrate specificity, and neuraminidase inhibitor sensitivity with the corresponding influenza B virus NAs. Especially the similar temperature profiles of WSEIV and influenza B virus NAs are interesting since the expectation was that the WSEIV NA would be more active at lower temperatures as found in the habitat of the lesser spiny eel. This also raises the possibility that the WSEIV is actually of mammalian or avian origin. Additional studies are needed to further determine if the virus is a *bona fide* fish virus or if it originated from other, warm-blooded animals.

The abundant expression of GM2 across different organs in zebrafish supports the possibility that the WSEIV could infect other species of fish as well. Consequently, human infection could occur with individuals directly interacting with these aquatic species at, e.g. at commercial fisheries or aquaria as well as during recreational fishing or swimming. The lesser spiny eel (*M. aculeatus*) and other *Macrognathus* species are distributed throughout Southeast Asia and are farmed and harvested for food^49^. Of note, GM2 is of course also present in humans. In addition, we found very limited antigenic similarity between WSEIV and influenza B virus glycoproteins. Cross-reactivity of mAbs was limited to a small subset of antibodies and no cross-reactivity was found in human serum suggesting that humans are immunologically naïve to the WSEIV glycoproteins.

In summary, the WSEIV HA and NA proteins show varying degrees of similarity to their influenza B virus counterparts. The HA displays sialic acid binding activity specifically towards GM2 and thereby differs substantially from known influenza A and B virus HAs, and the NA is indeed a sialidase with very similar functionality compared to influenza B virus NA. The data provided in this study contribute to our overall understanding of influenza B and influenza B-like viruses, and to understanding of the pandemic potential of the influenza B-like viruses from non-human reservoirs.

## Methods

### Cells and proteins

Human embryo kidney 293T cells were cultured in complete Dulbecco’s modified Eagle medium (DMEM; Life Technologies) constituted by DMEM supplemented with Pen-Strep antibiotics (100 U/ml penicillin, 100 µg/ml streptomycin; Gibco), 10% fetal bovine serum (FBS, HyClone), and 10 ml of 1 M 4-(2-hydroxyethyl)-1-piperazineethanesulfonic acid (HEPES, Life Technologies). Sf9cells (ATCC CRL-1711) and High Five cells (BTI-TN-5B1-4 subclone; Vienna Institute of Biotechnology) were grown in *Trichoplusia ni* medium-formulation Hink (TNM-FH) insect medium (Gemini Bioproducts) supplemented with Pen-Strep and 10% FBS, and serum free medium (SFM) insect cell medium (HyClone), respectively^33^.

The recombinant proteins used in this study (WSEIV HA, WSEIV NA, B/Malaysia/2506/2004 HA, B/Malaysia/2506/2004 NA, A/Vietnam/1203/2004 H5 HA, A/flat-faced bat/Peru/033/2010 H18 HA, A/Anhui/1/2013 N9 NA) were expressed and purified from High Five cell culture supernatant as described in detail previously^16^.

### Phylogenetic and comparative sequence analysis

Phylogenetic trees for the HA and NA were generated as described previously^33^. Briefly, sequences were obtained from the Global Initiative on Sharing All Influenza Data (GISAID), aligned using Clustal Omega (v1.2.4), and the phylogenetic trees were generated using FigTree (v1.4.3). The annotation of the tree was carried out in Adobe Illustrator CS5.1. Pairwise alignment of the WSEIV HA and NA (MG600041.1 and MG600042.1) and B/Malaysia/2506/2004 HA and NA (CY040449.1 and CY040451.1) was performed using Clustal Omega v1.2.4, following which the features of note were labeled in Adobe Illustrator CS5.1. For the rendered model of glycoproteins displaying sequence conservations, the WSEIV HA and NA were aligned pairwise against the B/Brisbane/60/2008 HA and NA. The alignment was superimposed on the B/Brisbane/60/2008 HA and NA structures publicly available on PDB (HA: 4FQM^50^; NA: 4CPL^51^) using UCSF Chimera.

### SDS–PAGE and Western blotting

Recombinant proteins (10 µg) were applied to 4–20% gradient polyacrylamide gels (Bio-Rad) after heating them for 20 min at 95 °C in 2× Laemmli buffer with 2% β-mercaptoethanol (BME). SDS–PAGE was performed at 200 V for 35 min following which the gels were stained with SimplyBlue Safe Stain (Thermo Fisher) to visualize the bands alongside a color prestained protein broad range standard (New England Biolabs). Deglycosylation was carried out using the Protein Deglycosylation Mix II (New England Biolabs) as per the manufacturer’s instructions.

Western blotting procedures to determine the proteolytic cleavage of the HA were carried out as previously described^24,33^. HEK293T cells were co-transfected with pCAGGS expression plasmids encoding for the corresponding HA and pcDNA3.1 plasmids encoding human airway proteases (Genscript). The Western blotting procedure was carried out with cell lysates, probing with either polyclonal sera raised against the WSEIV HA in female BALB/c mice (at a dilution of 1:100) or with a pool of anti-influenza B virus HA mAbs (at 10 µg/ml) characterized in this ref. ^33^.

### Hemagglutination assay

Recombinant HA starting at 10 µg diluted serially 2-fold was incubated with 0.5% chicken or turkey erythrocyte suspension and incubated at 4 °C for an hour. The plates were then scanned to determine the extent of hemagglutination of these erythrocytes.

### Glycan array

Glycan array binding analysis of the rHAs was carried out as described here^52,53^. Briefly, recombinant hexahistidine-tagged HA was precomplexed with a mouse anti-his Alexa 647 (antibody mouse anti-his 647 antibody, Abcam, ab237337, clone EPR20547) and goat-anti-mouse Alexa 647 antibodies (Thermo, Cat # A28181; RRID: AB_2536165). This was done in 50 µl PBS-T (phosphate-buffered saline with 0.1% Tween-20) in a 4:2:1 molar ratio, incubated for 15 min on ice, and the applied on the array for 90 min in a humidified chamber. Following multiple washes with PBS-T, PBS, and deionized water the arrays were scanned to detect HA binding. Mean RFU and standard deviation values were imported into Prism 7.0 and the corresponding graph was generated.

### Bio-layer interferometry

As described previously, biolayer interferometry with an Octet Red96 instrument (ForteBio) was used to determine the dissociation constant of the HA-GM2 receptor interaction^54^. Recombinant hexahistidine-tagged HAs at 10 µg/ml was loaded onto Ni-NTA biosensors (Fortebio) for 780 s to ensure saturation after a baseline step was established for 60 s. A second baseline was established post-loading spanning 120 s. Then the association (300 s) and dissociation (900 s) kinetics were recorded as the HA-loaded sensors were dipped into 1.5-fold serially diluted concentrations of recombinant GM2 (Sigma Aldrich). The reaction was carried out in a 1× kinetics buffer comprising of 1× PBS, 0.01% bovine serum albumin (BSA) and 0.002% Tween 20. The dissociation constant was calculated accordingly using the suitable model for a biphasic association and dissociation profile, and global curve fit was applied to all the sensors. Octet Red96 Data Acquisition v12.0 software was used to acquire the biolayer-interferometry data.

### Fusion assay

A polykaryon formation assay to determine the fusogenicity of the WSEIV HA was performed as described in detail previously^24^. HeLa cells (CCL-2, ATCC) were seeded in 96-well tissue culture plates (Corning) and transfected with a pCAGGS plasmid expressing the WSEIV HA. The following day, the cells were washed with Dulbecco’s phosphate buffer saline (DPBS), exposed to TPCK-treated trypsin at 5 µg/ml for 15 min, washed with DPBS, and subsequently exposed to pH adjusted DPBS ranging from pH 4.5 to 5.9 adjusted with citric acid. After 15 min of an acid pulse, the cells were washed and allowed to recover and fuse in complete DMEM for 4 h. The cells were then fixed with 3.7% paraformaldehyde in PBS and permeablized with 0.1% Triton X-100 for 15 min each. All of these steps were performed at 33 °C. Staining was performed using the HCS Cell Mask Green (Life Technologies) as per manufacturer’s instructions for 30 min at 5 µg/ml. Imaging was performed using EVOS FL Cell Imaging microscope (Thermo Fisher) and the images were collectively processed using Adobe Photoshop and annotated using Adobe Illustrator.

### ELLAs

ELLAs were performed as described in detail previously to determine the enzymatic activity of the NAs or the NA inhibitor sensitivity of the NAs^55^. Briefly, Immulon 4 HBX plates (Thermo Scientific) were coated with 100 µl/well of fetuin (Sigma) at 25 µg/ml overnight at 4 °C. The fetuin-coated plates were incubated with 2 fold dilutions of recombinant NAs starting at 10 µg/ml in 5% BSA in PBS overnight at the indicated temperatures. The following day, the plates were washed three times with PBS-T and were incubated with 5 µg/ml of peanut agglutinin conjugated to horseradish peroxidase (PNA-HRP) for two hours at room temperature. The plates were washed with PBS-T three times and developed with 100 µl of Sigmafast *o*-phenylenediamine dihydrochloride (OPD) (Sigma Aldrich). The only applied variation was that the overnight incubation was carried out at four different temperatures (4, 20, 33, and 37 °C) to determine the temperature-dependent profile of the NAs. When oseltamivir, zanamivir, and peramivir were used, the starting concentration applied was 156.28 µM with 2-fold serial dilutions, and they were pre-incubated with the recombinant NAs for 1 h shaking at 37 °C after which the conventional ELLA protocol was followed. Substrate specificity characterization and specific enzyme activity determination was performed in ELLA assays identical to that described in ref. ^26^. Absorbance measurements were carried out at 490 nm following the development with Sigmafast OPD, and the specific enzyme activity (inverse of half-maximum lectin binding) was determined following a non-linear regression analysis in Graphpad Prism 7, and was plotted normalized to the fetuin-ECA to determine activity per amount of protein in context of cleavage of α2,3 or α2,6-linked sialic acids.

### Michaelis–Menten kinetics

Enzyme kinetics and the Michaelis–Menten parameters were determined as described previously^32^. Briefly, recombinant NAs at a fixed concentration of 10 µg/ml were incubated with 1.5 fold dilutions of the fluorogenic MUNANA substrate in MES (2-ethanesulfonic acid2-ethanesulfonic acid) buffer with suitable blank controls for background fluorescence. The plates were incubated at 37 °C and readings for relative fluorescence units (RFUs) were recorded at every 90 s for 40 min using a Gen5 v3.0 Software and a Synergy H1 Microplate Reader (BioTek). The RFU readings were captured at excitation and emission wavelengths of 360 and 448 nM. Velocity of the reaction was determined by plotting the RFU readings against time, and the Michaelis–Menten parameters *V*_max_ and K_m_ were determined through non-linear regression fits of the velocity and MUNANA concentrations in Graphpad Prism 7.

### Free sialic acid assay and thin layer chromatography

Recombinant GM2 (100 μM) was incubated with WSEIV NA or BSA at 10 μg/ml. As a positive control, fetuin (100 μM) was incubated with WSEIV NA (10 μg/ml). The reactions were then analyzed using a Sialic Acid (NANA) assay kit (Abcam) as per the manufacturer’s instructions. The amount of free sialic acid was determined by extrapolation from a standard curve and graphed using Graphpad Prism 7. The same samples were also applied using a 700 series microliter syringe (Hamilton) onto pre-scored silica gel TLC plates (Millipore Sigma) and placed in a running buffer of ethanol:acetic acid:water at 5:2:1. As a control for both experiments, GM2 was also incubated with BSA as an irrelevant protein. Assay buffer without GM2 was also applied to the TLC plate as a blank control. The plates were visualized using a Bio-Rad Gel Doc (Bio-Rad), and the chromatogram was annotated on Adobe Illustrator.

### ELISAs

ELISAs were performed as previously described in detail^33^. Immulon 4 HBX plates (Thermo Scientific) were coated overnight with 2 µg/ml of recombinant protein in 1× coating buffer (Seracare). The plates were blocked with 3% milk powder in PBS-T for one hour at room temperature following which they were incubated with 3-fold dilutions of mAbs starting at 30 µg/ml or human serum samples at a dilution of 1:250 for two hours at room temperature. The plates were washed three times with PBS-T and stained with the appropriate goat anti-human (Invitrogen, 31410) or goat anti-mouse HRP-tagged (Rockland, 610-1302) antibody at a dilution of 1:3000 in PBS-T and developed using Sigmafast OPD. Information surrounding individual antibodies used in the primary staining procedure are available in the cited references.

### Human serum samples

Serum samples were obtained from healthy participants participating in a longitudinal, observation, non-interventional study (STUDY-16-01199, PI: V. Simon) approved by the Institutional Review Board of the Mount Sinai School of Medicine. Informed consent was obtained prior to study participation and participants provided written permission to biospecimen banking and future research use. Samples were collected between 2017 and 2019. All samples were de-identified and analyzed in a blinded manner.

### Reporting summary

Further information on research design is available in the Nature Research Reporting Summary linked to this article.

## Supplementary information

Reporting Summary

## Data Availability

All data are available in the manuscript or the supplementary materials. Source data are provided with this paper. HA (4FQM) and NA (4CPL) structures for B/Brisbane/60/2008 were obtained from PDB. Sequences for the HAs and NAs for the phylogenetic trees were obtained from GISAID (H1-H18: H1-H18: EPI1349891, EPI899625, EPI673678, EPI1007628, EPI942074, EPI1154383, EPI1090164, EPI1154159, EPI1103524, EPI953583, EPI774886, EPI1007631, EPI967018, EPI750076, EPI965435, EPI939704, EPI356309, EPI486922; B/Lee/1940 HA: EPI243230; B/Phuket/3073/2013 HA: EPI1799824; B/Colorado/06/2017 HA: EPI969380; N1-N11: EPI1381203, EPI899627, EPI939823, EPI1154448, EPI1007658, EPI939830, EPI750078, EPI941550, EPI965439, EPI356311, EPI356298; B/Lee/1940 NA: EPI366432; B/Phuket/3073/2013 NA: EPI1799823; B/Colorado/06/2017 NA: EPI969379). WSEIV HA and NA (MG600041.1 and MG600042.1) and B/Malaysia/2506/2004 HA and NA (CY040449.1 and CY040451.1) sequences were used for the pairwise alignment represented in Fig. 1. Source data are provided with this paper.

## Source data

Source Data
