# Supplementary material for: Functionality of the putative surface glycoproteins of the Wuhan spiny eel influenza virus
Source: Nat Commun. 2021 Oct 25;12:6161. doi: 10.1038/s41467-021-26409-2 (PMC8546056; doi:10.1038/s41467-021-26409-2)
Supplement: Supplementary file 1 — Reporting Summary [file 41467_2021_26409_MOESM1_ESM.pdf]

nature portfolio

Corresponding author(s): Florian Krammer

Last updated by author(s): Sep 6, 2021

Reporting Summary

Nature Portfolio wishes to improve the reproducibility of the work that we publish. This form provides structure for consistency and transparency in reporting. For further information on Nature Portfolio policies, see our Editorial Policies and the Editorial Policy Checklist.

Statistics

For all statistical analyses, confirm that the following items are present in the figure legend, table legend, main text, or Methods section.

n/a	Confirmed	
☐	☒	The exact sample size (*n*) for each experimental group/condition, given as a discrete number and unit of measurement
☐	☒	A statement on whether measurements were taken from distinct samples or whether the same sample was measured repeatedly
☐	☒	The statistical test(s) used AND whether they are one- or two-sided<br>*Only common tests should be described solely by name; describe more complex techniques in the Methods section.*
☒	☐	A description of all covariates tested
☐	☒	A description of any assumptions or corrections, such as tests of normality and adjustment for multiple comparisons
☐	☒	A full description of the statistical parameters including central tendency (e.g. means) or other basic estimates (e.g. regression coefficient) AND variation (e.g. standard deviation) or associated estimates of uncertainty (e.g. confidence intervals)
☒	☐	For null hypothesis testing, the test statistic (e.g. *F*, *t*, *r*) with confidence intervals, effect sizes, degrees of freedom and *P* value noted<br>*Give P values as exact values whenever suitable.*
☒	☐	For Bayesian analysis, information on the choice of priors and Markov chain Monte Carlo settings
☒	☐	For hierarchical and complex designs, identification of the appropriate level for tests and full reporting of outcomes
☒	☐	Estimates of effect sizes (e.g. Cohen's *d*, Pearson's *r*), indicating how they were calculated

Our web collection on statistics for biologists contains articles on many of the points above.

Software and code

Policy information about availability of computer code

Data collection	Absorbance data was collected using BioTek Gen5 v3.0 software. Octet Red96 Data Acquisition software v12.0 was used to obtain biolayer-interferometry data.
Data analysis	Data analysis was carried out using Prism 7.0 to generate the graphs represented in the manuscript. Clustal Omega v.1.2.4 was used to analyze and align sequence data. Figtree v1.4.3 was used to generate the phylogenetic trees.

For manuscripts utilizing custom algorithms or software that are central to the research but not yet described in published literature, software must be made available to editors and reviewers. We strongly encourage code deposition in a community repository (e.g. GitHub). See the Nature Portfolio guidelines for submitting code & software for further information.

Data

Policy information about availability of data

All manuscripts must include a data availability statement. This statement should provide the following information, where applicable:

- Accession codes, unique identifiers, or web links for publicly available datasets
- A description of any restrictions on data availability
- For clinical datasets or third party data, please ensure that the statement adheres to our policy

All data are available in the manuscript or the supplementary materials. Source data are provided with this paper.
HA (4FQM) and NA (4CPL) structures for B/Brisbane/60/2008 were obtained from PDB. Sequences for the HAs and NAs for the phylogenetic trees were obtained from GISAID (H1-H18: H1-H18: EPI1349891, EPI899625, EPI673678, EPI1007628, EPI942074, EPI1154383, EPI1090164, EPI1154159, EPI1103524, EPI953583, EPI774886, EPI1007631, EPI967018, EPI750076, EPI965435, EPI939704, EPI356309, EPI486922; B/Lee/1940 HA: EPI243230; B/Phuket/3073/2013 HA: EPI1799824; B/Colorado/06/2017 HA: EPI969380; N1-N11: EPI1381203, EPI899627, EPI939823, EPI1154448, EPI1007658, EPI939830, EPI750078, EPI941550, EPI965439,

EPI356311, EPI356298; B/Lee/1940 NA: EPI366432; B/Phuket/3073/2013 NA: EPI1799823; B/Colorado/06/2017 NA: EPI969379). WSEIV HA and NA (MG600041.1 and MG600042.1) and B/Malaysia/2506/2004 HA and NA (CY040449.1 and CY040451.1) sequences were used for the pairwise alignment represented in Fig. 1.

Field-specific reporting

Please select the one below that is the best fit for your research. If you are not sure, read the appropriate sections before making your selection.

- [x] Life sciences
- [ ] Behavioural & social sciences
- [ ] Ecological, evolutionary & environmental sciences

For a reference copy of the document with all sections, see nature.com/documents/nr-reporting-summary-flat.pdf

Life sciences study design

All studies must disclose on these points even when the disclosure is negative.

Sample size	For most experiments, recombinant WSEIV glycoproteins were run against their comparative controls detailed in the study. For human sera ELISAs, 18 serum samples obtained from the Personalized Virology Initiative at the Icahn School of Medicine at Mount Sinai were used against 5 recombinant glycoproteins in ELISAs detailed in the study. No sample size calculation was performed, sample size was chosen based on availability. For direct comparison of HA or NA proteins, a relevant influenza B virus was chosen for qualitative comparison. For probing with mAbs or human samples, all available mAbs human samples at the time of the study were used.
Data exclusions	No data was excluded.
Replication	All experiments contained at least technical duplicates. Experimental findings were reliably reproduced. Suitable positive and negative controls were used appropriately in every experiment detailed in the study. WSEIV HA-GM2 binding was identified using a glycan microarray and validated using biolayer-interferometry. WSEIV NA cleavage of GM2 was validated with a free sialic acid detection assay and thin-layer chromatography.
Randomization	No randomization was performed. Randomization would have overly complicated the study and would have added a quite complex system and additional study personnel, leading to a higher chance of human error. In addition, as this was an exploratory study which contrasts two different viruses we had little concern about a bias by the study personnel.
Blinding	No blinding was performed. Blinding would have overly complicated the study and would have added a quite complex system and additional study personnel, also leading to a higher chance of human error. In addition, as this was an exploratory study which contrasts two different viruses we had little concern about a bias by the study personnel.

Reporting for specific materials, systems and methods

We require information from authors about some types of materials, experimental systems and methods used in many studies. Here, indicate whether each material, system or method listed is relevant to your study. If you are not sure if a list item applies to your research, read the appropriate section before selecting a response.

Materials & experimental systems

n/a	Involved in the study	
[ ]	[x]	Antibodies
[ ]	[x]	Eukaryotic cell lines
[x]	[ ]	Palaeontology and archaeology
[x]	[ ]	Animals and other organisms
[ ]	[x]	Human research participants
[x]	[ ]	Clinical data
[x]	[ ]	Dual use research of concern

Methods

n/a	Involved in the study	
[x]	[ ]	ChIP-seq
[x]	[ ]	Flow cytometry
[x]	[ ]	MRI-based neuroimaging

Antibodies

Antibodies used	mAbs used:<br>CR9114 and CR8033 (human)<br>A05, 3C02, E04 (human)<br>1B5, 1D2, 2H10, 3A10, 3H10, 4C4, 4C10, 5C5, 7C7, 8A5, 8G3, 8G12, 9B9, 9C6, 11C12, 12F12 (murine)<br>3A7, 3F4, 3D7, 4C2 (murine)<br>1G01 (human)<br>1A03, 1D05, 1G05, 2D10, 2E01, 2H09, 3C01 (human)<br>1F2, 1F4, 3G1, 4B2, 4F11 (murine)<br>Starting concentrations for these mAbs in ELISA are specified in the figures.

	polyclonal serum used: anti WSEIV HA mouse serum<br>All listed antibodies were produced at the Krammer laboratory<br><br>Secondary antibodies used in ELISAs and Western blots are described in the manuscript. They are as follows: mouse anti-his 647 antibody (Abcam, ab237337, clone EPR20547); goat -anti-mouse Alexa 647 (Thermo, Cat # A28181; RRID: AB_2536165); Goat anti-human IgG H+L HRP-tagged (Invitrogen, 31410); Goat anti-mouse IgG H+L HRP-tagged (Rockland, 610-1302).
Validation	All primary antibodies and mAbs used have been characterized (which can be interpreted as 'qualified') in previous studies. Validation in the technical sense of the word has not been performed for any of the mAbs (validation - which is a big word and often misunderstood - is typical not done for reagents until used for Phase III human trials).<br><br>CR9114 and CR8033 (human) have been described and characterized in https://pubmed.ncbi.nlm.nih.gov/22878502/<br>A05, 3C02, E04 (human) have been described and characterized in https://pubmed.ncbi.nlm.nih.gov/32907980/<br>1B5, 1D2, 2H10, 3A10, 3H10, 4C4, 4C10, 5C5, 7C7, 8A5, 8G3, 8G12, 9B9, 9C6, 11C12, 12F12 (murine) have been described and characterized in https://pubmed.ncbi.nlm.nih.gov/30626682/<br>3A7, 3F4, 3D7, 4C2 (murine) have been characterized internally<br>1G01 (human) has been described and characterized in https://pubmed.ncbi.nlm.nih.gov/31649200/<br>1A03, 1D05, 1G05, 2D10, 2E01, 2H09, 3C01 (human) have been described and characterized in https://pubmed.ncbi.nlm.nih.gov/32976769/<br>1F2, 1F4, 3G1, 4B2, 4F11 (murine) have been described and characterized in https://pubmed.ncbi.nlm.nih.gov/28827718/<br>polyclonal serum used: anti WSEIV HA mouse serum, characterized in house<br><br>Validation for the commercially available secondary antibodies were obtained on the aforementioned manufacturer's websites for the corresponding applications of Western Blots or ELISAs. No additional validation was performed upon receipt of the commercial antibody.

Eukaryotic cell lines

Policy information about cell lines

Cell line source(s)	HeLa, HEK293T, and Sf9 cells were sourced from ATCC (CCL-2, CRL-3216, CRL-1711). BTI-TN-5B1-4 are an original clone that was provided from the Boyce Thompson Institute to the University of Natural Resources and Life Sciences, Vienna and from there to the Icahn School of Medicine at Mount Sinai.
Authentication	Cell lines (except BTI-TN-5B1-4) were obtained from a commercial source. After they were received, cells were recovered and not further authentication was performed.
Mycoplasma contamination	Cell lines tested negative for mycoplasma contamination.
Commonly misidentified lines (See ICLAC register)	No commonly misidentified cell lines were used.

Human research participants

Policy information about studies involving human research participants

Population characteristics	The samples used are deidentified samples taken between 2017 and 2019 from a longitudinal cohort of adults who are monitored for immune responses to influenza virus vaccination. No additional information is available.
Recruitment	No subjects were recruited for this study. Deidentified pre-existing samples were used.
Ethics oversight	The longitudinal study was approved by the Mount Sinai IRB under number STUDY-16-01199 (PI: V. Simon). All participants provided informed consent.

Note that full information on the approval of the study protocol must also be provided in the manuscript.
